# The Effect of GLUT1 and HIF-1α Expressions on Glucose Uptake and Patient Survival in Non-Small-Cell Lung Carcinoma

**DOI:** 10.3390/ijms241310575

**Published:** 2023-06-24

**Authors:** Josipa Kokeza, Ante Strikic, Marin Ogorevc, Nela Kelam, Martina Vukoja, Ivo Dilber, Sandra Zekic Tomas

**Affiliations:** 1 Department of Pulmonology, University Hospital of Split, Spinčićeva 1, 21000 Split, Croatia; josipa.kokeza@gmail.com; 2 Department of Oncology and Radiotherapy, University Hospital of Split, Spinčićeva 1, 21000 Split, Croatia; astrikic@gmail.com; 3 Department of Anatomy, Histology and Embryology, University of Split School of Medicine, Šoltanska 2, 21000 Split, Croatia; marin.ogorevc2@gmail.com (M.O.); nela.kelam@mefst.hr (N.K.); 4 Laboratory of Morphology, Department of Histology and Embryology, School of Medicine, University of Mostar, 88 000 Mostar, Bosnia and Herzegovina; martina.vukoja@mef.sum.ba; 5 Department of Oncology and Nuclear Medicine, General Hospital Zadar, Ul. Bože Peričića 5, 23000 Zadar, Croatia; ivodilber81@gmail.com; 6 Department of Pathology, Forensic Medicine and Cytology, University Hospital of Split, Spinčićeva 1, 21000 Split, Croatia; 7 Department of Pathology, University of Split School of Medicine, Šoltanska 2, 21000 Split, Croatia

**Keywords:** GLUT1, HIF-1α, lung adenocarcinoma, lung squamous cell carcinoma, PET CT

## Abstract

Lung cancer is the second-most-common cancer while being the leading cause of cancer deaths worldwide. It has been found that glucose transporter 1 (GLUT1) and hypoxia-inducible factor 1α (HIF-1α) are overexpressed in various malignancies and that they correlate with the maximum standard uptake values (SUVmax) on 18F-fluorodeoxyglucose-positron emission tomography/computed tomography (18F-FDG PET/CT) and poor prognosis. In this study, we aim to evaluate the relationship between the SUVmax, GLUT1, and HIF-1α expression with primary tumor size, histological type, lymph node metastases, and patient survival. Of the 48 patients with non-small-cell lung cancer, those with squamous cell carcinomas (SCCs) had significantly higher GLUT1 and HIF-1α immunohistochemical expressions in comparison to adenocarcinomas (ACs), while there was no statistically significant difference in FDG accumulation between them. No significant correlation was noted between either GLUT1 or HIF-1α protein expression and FDG uptake and overall survival. However, an analysis of tumor transcriptomics showed a significant difference in overall survival depending on mRNA expression; patients with SCC and high *HIF-1α* levels survived longer compared to those with low *HIF-1α* levels, while patients with AC and low *GLUT1* levels had a higher average survival time than those with high *GLUT1* levels. Further studies are needed to determine the prognostic value of the expression of these factors depending on the histologic type.

## 1. Introduction

Lung cancer (LC) is estimated to be the leading cause of cancer mortality in 2023, while being the second-most-common cancer diagnosed in men and women in the US, after prostate and breast cancers, respectively [1]. A prompt diagnosis and the early initiation of treatment are crucial in LC management. After an initial diagnostic evaluation, a computed tomography (CT) scan of the chest and upper abdomen should be performed. In patients that can undergo surgery, a CT scan with positron emission tomography (PET) is part of the basic diagnostic evaluation and should be followed by tumor biopsy, staging, and functional evaluations [2,3,4].

According to the World Health Organization (WHO), lung tumors are classified into epithelial tumors, pulmonary neuroendocrine neoplasms, ectopic tissue tumors, lung-specific mesenchymal tumors, and hematolymphoid tumors, with epithelial tumors being the most common [5]. Based on the histology, LC is traditionally divided into small-cell lung carcinomas (SCLCs) and non-small-cell lung carcinomas (NSCLCs) [6]. Among NSCLCs, adenocarcinomas (ACs) and squamous cell carcinomas (SCCs) are the most common [7] and are the focus of the present study.

At the beginning of the 20th century, Otto Warburg described the phenomenon in which malignant cells, even with sufficient oxygen concentration, prefer glycolysis as the main pathway for ATP production. Contrary to the above, normal cells metabolize glucose for energy production mainly through oxidative phosphorylation and through glycolysis, only under anaerobic conditions [8,9]. Since the main function of glucose transporters is to enable the constant availability of glucose to cells, and they conduct this by controlling the transfer of glucose between the intracellular and extracellular parts, it is clear that they mediate the Warburg phenomenon [10].

Glucose transporter 1 (GLUT1) is a transmembrane protein involved in passive glucose transport and it is expressed on numerous cell types [11,12]. It has a high affinity for glucose, compared to other glucose transporters, and is therefore of great importance in tissues where glucose is the main energy source [13]. It also plays a significant role in the transport of fluorodeoxyglucose (FDG) used in the detection of metabolically active lesions by means of PET CT [14,15,16]. GLUT1 was found to be overexpressed in various solid and hematological malignancies, such as diffuse large B-cell lymphomas, colorectal carcinomas, hepatocellular carcinomas, head and neck cancers, gastrointestinal stromal tumors, sarcomas, and pancreatic and lung cancers [17,18,19,20,21,22,23,24], and a study by Higashi et al. has shown a correlation between GLUT1 expression and FDG accumulation in human cancer cells [25]. Unfortunately, the results of the studies are ambiguous in regard to the correlation of GLUT1 expression with the grade and stage of the tumor, as well as the clinical outcome [12,22,26].

Hypoxia is a hallmark of the tumor microenvironment present in the majority of solid tumors and is associated with poor prognosis [27,28]. Under hypoxic conditions, cell proliferation is reduced, however, cancer cell mutations and genetic alterations together with highly dynamic metabolic reprogramming allow for cell proliferation, despite the low-oxygen concentration [29]. Hypoxia-inducible factor 1α (HIF-1α) plays a major role in the cellular response to hypoxia by regulating the expression of many genes involved in adaptive processes that allow cell survival under low oxygen conditions [30]. It regulates several aspects of tumorigenesis, including tumor angiogenesis, cell proliferation, metabolism, metastasis, differentiation, and the response to radiotherapy [31,32]. HIF-1α is overexpressed in several cancers, such as colon, kidney, pancreas, esophagus, endometrial, prostate, breast, stomach, and lung cancers [33,34,35,36]. Some studies have suggested that HIF-1α may be prognostic in lung cancer, as it could be associated with tumor aggression and related to chemo-resistance [37,38,39]. Studies showed that patients with lung cancer and high HIF-1α expression have a significantly shorter overall survival (OS) and that high HIF-1α expression is also associated with tumor metastasis and a higher Eastern Cooperative Oncology Group (ECOG) rating [40].

It has been shown that the dysregulation of the phosphatidylinositol 3-kinase (PI3K)/AKT/mammalian target of the rapamycin (mTOR) signaling pathway, which is one of the key factors in the tumorigenesis of various malignancies, leads to GLUT1 and HIF-1α overexpressions, thus allowing these factors to influence tumor metabolism and cancer propagation [41]. The recently published study by Bao et al. demonstrated the increase in the radiosensitivity of laryngeal carcinomas by the double knock-out of *HIF-1α* and *GLUT1*, which leads to reduced PI3K/Akt/mTOR signaling [42]. Their results showed that the simultaneous action of HIF-1α and GLUT1 with the PI3K/Akt/mTOR signaling pathway facilitated cell viability and glucose uptake during radiotherapy under hypoxic conditions.

The aim of the present study is to determine the protein expression levels of GLUT1 and HIF-1α in the ACs and SCCs of the lungs and to correlate the findings with PET CT scanning results. Additionally, we perform an analysis of the transcriptomic data to determine whether the mRNA expression of the two factors is correlated to patient survival. Our findings can elucidate the roles of GLUT1 and HIF-1α in the glucose metabolism of the most common NSCLCs and determine whether it is a potential prognostic marker.

## 2. Results

Our study included a total of 48 patients: 32 men (66.66%) and 16 women (33.33%). The average age of all the patients was 65 years old, precisely 66 years old for men and 63 years old for women. AC was diagnosed in 34 cases and the rest were SCC cases. Lymph node metastases were histologically confirmed in 16 patients, while 30 patients had no metastatic tissue in their lymph nodes, and for 2 patients the information was unavailable. Of the 16 histologically confirmed lymph node metastases, 13 were AC and 3 were SCC. There was no statistically significant difference between ACs and SCCs regarding the sex and patients’ age, as well as the tumor stage. OS was longer in AC patients (64 ± 41 months) compared to SCC patients (38 ± 35 months), but without statistical significance. There was no statistically significant difference in disease-free survival (DFS) between AC (25 ± 19 months) and SCC (18 ± 18 months) patients. The average size of the primary process in AC was 42.3 mm, compared to 39.8 mm in SCC. Analyzing the size of the primary tumor with histologically determined positive (50.4 ± 22.8 mm) and negative lymph (38.7 ± 22.12 mm) node metastases; it was observed that metastasized tumors were larger, but without statistical significance.

### 2.1. GLUT1 Protein Expression

**Figure 1:**
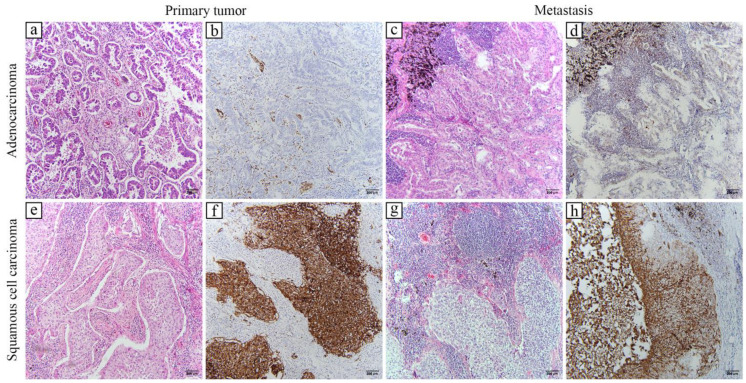
Morphology and GLUT1 expression in lung carcinoma. Typical glandular morphology is observed in both primary (**a**) and metastatic lesions (**c**) of lung adenocarcinomas. Weak-to-no GLUT1 expression is seen in the primary adenocarcinoma lesion (**b**), while a mild, cytoplasmatic expression characterizes the metastasis (**d**). Morphology typical for squamous cell carcinomas is visible in primary (**e**) and metastatic lesions (**g**). Both lesions display strong, membranous GLUT1 expression in most of their tumor cells (**f**,**h**). Hematoxylin and eosin staining (**a**,**c**,**e**,**g**); immunohistochemical staining to GLUT1 (**b**,**d**,**f**,**h**). All images are taken at ×100 magnification; scale bars represent 200 µm.

The tumors included in the study were classified according to the WHO Classification of Lung Tumors. All of the ACs were invasive non-mucinous ACs with a histologic growth pattern in keeping with acinar, papillary, micropapillary, and solid ACs. SCCs ranged from well-differentiated keratinized SCCs to poorly differentiated SCCs. GLUT1 expression was determined according to the intensity of membranous and/or cytoplasmatic brown staining of tumor cells as weak, moderate, or strong in comparison to the GLUT1 expression in red blood cells that served as a positive control. The pattern of GLUT1 staining was either focal or diffuse; however, most of the tumors had a heterogeneous staining pattern (Figure 1).

GLUT1 immunohistochemical expression determined with the histochemical scoring assessment (HScore) method in primary tumors and metastatic lymph nodes was compared between the clinical characteristics of patients, tumor stage, and histological subtype (Table 1). SCC had a significantly higher GLUT1 immunohistochemical expression in both primary tumors and lymph node metastasis in comparison to AC (*p* < 0.0001, *p* = 0.0196, respectively). There was no statistically significant difference in the primary tumor and lymph node GLUT1 immunohistochemical expression regarding sex, tumor stage, and lymph node positivity according to PET CT scans.

A statistically significant correlation was noted between primary tumor GLUT1 protein expression and GLUT1 expression in corresponding lymph node metastases (*p* = 0.0078). GLUT1 expressions of primary tumors and lymph nodes were in positive, but statistically insignificant, correlations with the maximum standard uptake values (SUVmax) of FDG in primary tumors and lymph nodes, respectively (*p* = 0.0670, *p* = 0.1947). There was no statistically significant correlation between GLUT1 protein expression and primary tumor size, OS, and DFS (*p* = 0.9058, *p* = 0.5037, *p* = 0.8959, respectively).

### 2.2. HIF-1α Protein Expression

**Figure 2:**
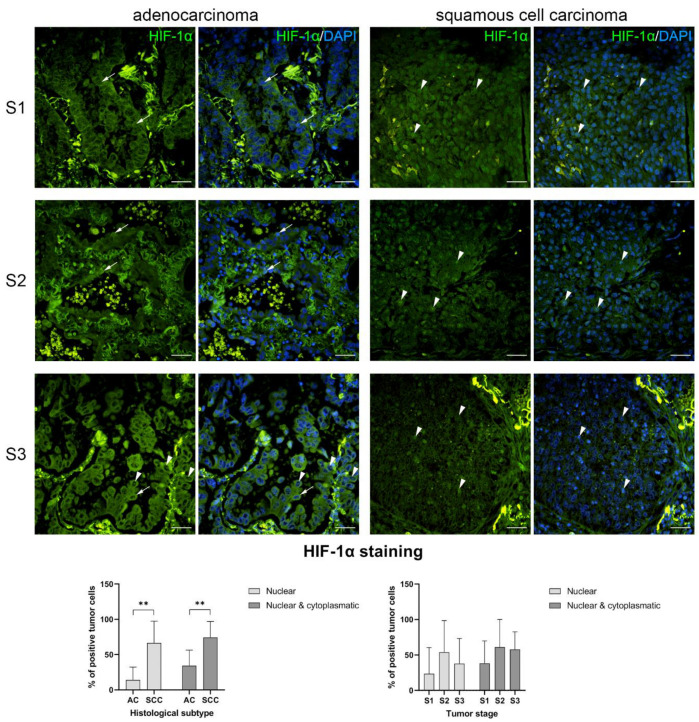
HIF-1α protein expression in lung carcinoma. S1—stage I, S2—stage II, S3—stage III. Some cells of lung adenocarcinoma lesions display cytoplasmatic HIF-1α staining (arrows), while only a few cells display nuclear staining (arrowheads). Conversely, most cells of lung squamous cell carcinomas demonstrate strong nuclear HIF-1α staining (arrowheads). Immunofluorescent staining to HIF-1α; images are taken at ×400 total magnification; scale bars represent 100 µm. Graphs represent the mean value HIF-1α positive cells; error bars represent the standard deviation; ** *p* < 0.01.

HIF-1α protein expression was demonstrated by the green fluorescent signal, while blue DAPI staining characterized all cell nuclei (Figure 2). Red blood cells and accumulations of fibrous extracellular material showed green-to-yellow autofluorescence and represent artifacts of immunofluorescent staining. The majority of AC samples displayed weak-to-no cytoplasmatic HIF-1α staining, with only some cells having moderate-to-strong cytoplasmatic staining. Some AC samples had nuclear HIF-1α staining in their tumor cells; however, the staining was weak. When analyzing the SCC samples, strong nuclear HIF-1α staining was present in most of the tumor cells, while only some cells showed exclusively cytoplasmatic staining. There were no noticeable differences in HIF-1α staining between different tumor stages within the histological subtypes.

HIF-1α immunofluorescent expression in primary tumors was compared between the clinical characteristics of patients, tumor stage, and histological subtype (Table 2). SCC had a significantly higher percentage of HIF-1α-positive cells in comparison to AC, both when analyzing only nuclear (*p* = 0.0025) and nuclear and cytoplasmatic positivity (*p* = 0.0038). There was also a significantly higher percentage of HIF-1α-positive cells among tumors with positive lymph nodes on PET CT scans, compared to tumors with PET CT negative lymph nodes. This was true for both nuclear (*p* = 0.0121) and nuclear and cytoplasmatic positivity (*p* = 0.0071). There was no significant difference in HIF-1α protein nuclear expression regarding sex and tumor stage; however, there was a significant difference between male and female patients when analyzing nuclear and cytoplasmatic positivity (*p* = 0.0089).

There was a statistically significant positive correlation between only nuclear and nuclear and cytoplasmatic HIF-1α positivity in primary LC lesions (*p* < 0.0001); however, when analyzing ACs and SCCs separately, this was true for SCCs (*p* = 0.0004), but not ACs (*p* = 0.1939). There was a statistically significant positive correlation between both nuclear (*p* = 0.0392) and nuclear and cytoplasmatic (*p* = 0.0498) HIF-1α positivity and GLUT1 HScore. There was a weak negative, but nonsignificant, correlation between nuclear HIF-1α positivity and FDG SUVmax of primary tumors (*p* = 0.6165), and the same was true for nuclear and cytoplasmatic HIF-1α positivity (*p* = 0.7602). There was no statistically significant correlation between HIF-1α positivity and age, primary tumor size, OS, and DFS, for both nuclear and nuclear and cytoplasmatic positivity.

### 2.3. Gene Expression Analysis

**Figure 3:**
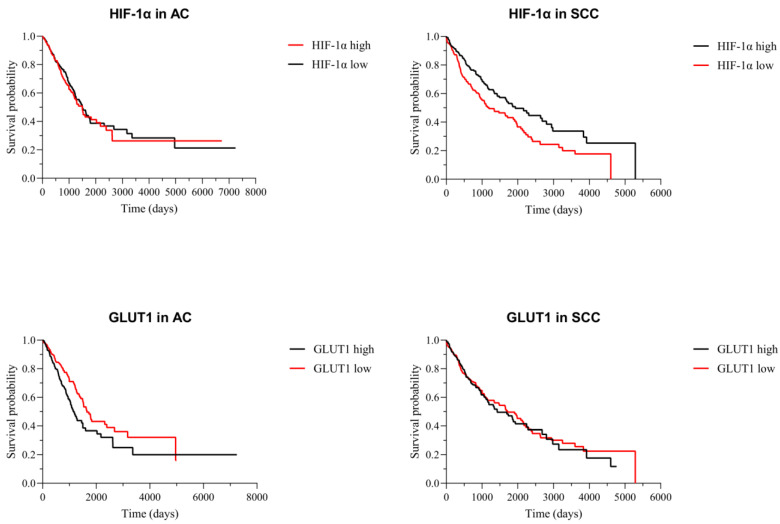
Graphic representation of survival analysis (days) of *HIF-1α* and *GLUT1* high (black line)- and low (red line)-mRNA-expression in lung ACs and SCCs. The Kaplan–Meier method and log-rank test were used for survival length. The data are used from the GDC TCGA Lung Adenocarcinoma (LUAD) and GDC TCGA Lung Squamous Cell Carcinoma (LUSC) studies.

*HIF-1α* and *GLUT1* mRNA expressions obtained from the Genomic Data Commons The Cancer Genome Atlas (GDC TCGA) Lung Adenocarcinoma (LUAD) and GDC TCGA Lung Squamous Cell Carcinoma (LUSC) studies were analyzed to determine the survival rate and average survival time (ast) between high- and low-expression groups (Figure 3). There was no statistically significant difference (*p* = 0.523) in survival time between *HIF-1α* high (ast 1498 days)- and low (ast 1499 days)-expression groups of lung ACs. There was a statistically significant difference (*p* = 0.0017) in survival time between *HIF-1α* high (ast 1933 days)- and low (ast 1190 days)-expression groups of lung SCCs. When analyzing *GLUT1* mRNA expression, there was a statistically significant difference (*p* = 0.0053) in survival time between high (ast 1171 days)- and low (ast 1653 days)-expression groups of lung ACs, while there was no significant difference (*p* = 0.7675) between high (ast 1426 days)- and low (ast 1713 days)-expression groups of lung SCCs.

### 2.4. PET CT Scans and FDG Uptake

PET CT scans (Vereos Digital PET/CT, Philips, Amsterdam, The Netherlands) detected only 25% of histologically confirmed lymph node metastases. Furthermore, out of the lymph nodes that were marked as potentially positive on PET CT scans, only one-third were histologically confirmed as metastatic. CT scans without PET detected 40% of histologically confirmed metastatic lymph nodes and 40% of lymph nodes marked as potentially positive on CT scans were confirmed positive by a histological analysis. The congruence between CT scans with and without PET was around 80%.

FDG accumulation expressed as SUVmax measured by PET CT scans was also compared between the clinical characteristics, histological subtype, and tumor stage of LC patients (Table 3). There was no statistically significant difference in the tumor and lymph node FDG accumulation regarding sex, the histological subtype of LC, or tumor stage. The SUVmax of primary lesions was higher in the cases of PET CT-positive lymph nodes; however, this difference was not statistically significant (*p* = 0.055).

There was a negative, but statistically insignificant, correlation between FDG uptake and OS (*p* = 0.1677), as well as DFS (*p* = 0.5571). There was, however, a significant positive correlation between FDG uptake and primary tumor size (*p* = 0.0043).

## 3. Discussion

The results of the presented study show a higher GLUT1 immunohistochemical expression in SCC primary tumors compared to AC. These findings are in accordance with the previous studies, although our study was the first one to use the semiquantitative HScore method for the GLUT1 immunohistochemical expression analysis of LCs [26,43,44,45,46,47,48]. Furthermore, we found a significantly higher GLUT1 immunohistochemical expression in SCC metastatic lymph nodes compared to AC lymph node metastases. On the contrary, FDG accumulation, although statistically insignificant, was higher in both primary AC and lymph nodes with AC metastases compared to SCC primary and metastatic tumors. To date, the results for FDG accumulation in regards to the LC histology subtype are equivocal; some studies showed higher FDG accumulation in SCCs and others showed no difference in FDG accumulation between various LC histology subtypes [49,50,51]. The possible explanation for such diverse study results can be found in the fact that FDG accumulation depends on the GLUT1 transporter. GLUT1 is mainly located on the membrane of tumor cells in SCCs, which facilitates the intake of FDG, unlike when GLUT1 is located in the cytoplasm of tumor cells, which can be the case in ACs [47]. A study by Khandani et al. found no correlation between FDG accumulation and GLUT1 positivity in LCs [52]. In our study, while FDG accumulation was in a positive correlation with GLUT1 HSCORE, not just in primary tumors, but also in metastatic lymph nodes, the results were not statistically significant. The discrepancy between our’s and other studies can be attributed to other factors that affect FDG uptake.

One of those factors is HIF-1α as it has been shown that FDG uptake is positively correlated with HIF-1α immunohistochemical expression [53]. Contrary to this study, we have not found a significant correlation between HIF-1α protein expression and FDG uptake; however, the primary tumors of patients with PET/CT-positive lymph nodes had a significantly higher HIF-1α protein expression compared to the patients with PET/CT-negative lymph nodes. The HIF-1α protein expression was significantly higher in SCCs compared to ACs, which is in line with the previous studies [54]. We also found a significant positive correlation between HIF-1α protein expression and GLUT1 HScore, as has been previously reported [53]. This is in accordance with the fact that HIF-1α has a role in the induction of GLUT1 expression, as well as other factors regulating numerous aspects of tumorigenesis, including angiogenesis, cell proliferation, metabolism, metastatic potential, and differentiation, but also response to radiotherapy [25,31,32,55,56].

Another factor affecting FDG uptake is tumor size. Higher SUVmax values are expected in tumors with larger volumes and cancer cell proliferation [57]. In our study, we also observed a significant positive correlation between SUVmax values and tumor size. A study by Xu et al. [53] analyzed the correlations between FGD uptake and GLUT1 protein expression, HIF-1α protein expression, and tumor size. While positive correlations with FDG uptake were found for all three factors, tumor size had the highest correlation coefficient. Taking this into account and considering that the ACs in our study were larger than the SCCs, the lack of a significant correlation between FDG accumulation and GLUT1 and HIF-1α expressions (which were significantly higher in SCCs) can be explained by the tumor size having a stronger contribution to FDG uptake.

It is important to point out that FDG accumulation, apart from malignant tumors, can also be seen in benign tumors due to the increased vascularization and permeability of blood vessels, and not an excessive expression of GLUTs [44,58]. As a result, PET CT with FDG has relatively low specificity and a false-positive finding posing as one of the major problems in the LC clinical staging, especially in regard to nodal staging [44,58]. In our study, CT proved to be of more diagnostic value than PET CT when it came to tumor staging, because 40% of histologically confirmed metastatic lymph nodes were detected via CT, compared to only one-third via PET CT. CT proved to be a more sensitive method with a higher positive predictive value compared to PET CT. Since PET CT is often used as a standard preoperative diagnostic method, this information can be valuable for the algorithm of the patient’s tumor-node-metastasis (TNM) staging.

FDG accumulation in combination with GLUT1 expression can provide a better insight into the prognosis of the disease. A study by Murakami et al. found a significant decrease in DFS in LC patients with a high SUVmax (with a cutoff at ≥2.15), but only in a subgroup of ACs [59]. Similarly, Shimizu et al. described significantly lower DFS for LC patients with ACs and a high SUVmax (cutoff at >3.95), but found no difference in patients with SCCs [60]. A meta-analysis by Tan et al. determined that OS was significantly lowered in NSCLC patients with high GLUT1 expressions (hazard ratio, HR = 2.21); however, after stratifying by ethnicity, no significant difference was found for Caucasian patients. The DFS was, however, significantly lower for patients with a high GLUT1 (HR = 1.73) and this remained true after stratification. The cutoff value for high GLUT1 expression was not described, nor were the patients stratified by tumor histologic type [48]. Our study showed that the GLUT1 protein expression and SUVmax of the primary tumor negatively correlated with OS and DFS, but without statistical significance. However, when analyzing the transcriptomic data from the GDC TCGA database, we found a significant decrease in OS among patients with a high *GLUT1* mRNA expression in their primary tumors, compared to those with a low *GLUT1* expression. This was true for ACs; however, no significant difference was found in SCCs. The opposite was true for *HIF-1α* mRNA expression as patients with SCCs and high levels of *HIF-1α* had a significantly higher OS rate compared to patients with low *HIF-1α* levels, while there was no significant difference for patients with ACs. Interestingly, a study by Swinson et al. determined a significant association between high HIF-1α positivity (>60% of positive tumor cells) and decreased median survival of NSCLC patients; however, when observing HIF-1α expression as a continuous variable, as we performed in our study, no significant association was found [54]. He et al. found a significant decrease in OS among NSCLC patients with high HIF-1α plasma levels (>297.7 pg/mL) for patients with SCCs; however, there was no significant difference after stratifying patients by TNM stage [61]. It is important to note that these studies analyzed protein, not mRNA, expression and that they had significantly fewer patients than the GDC TCGA database used in this study.

The main limitations of the study were its small sample size, retrospective nature, and possible selection bias due to all the samples being collected from only one center; however, it should be emphasized that our Pathology Department is a reference center for the region of Dalmatia (Croatia) and parts of the neighboring Republic of Bosnia and Herzegovina.

## 4. Materials and Methods

### 4.1. Tissue Procurement and Processing

This retrospective cross-sectional study was conducted in the Pathology Department, University Hospital Centre Split, Croatia, and was approved by the Hospital Ethics Committee. The LC samples were selected from the Pathology Department’s archive. The institute’s database of pathohistological reports was searched using the International Classification of Diseases (ICD)-10 code: C34, which stands for the malignant neoplasm of the bronchia and lung. The inclusion criteria were operational materials (lobectomies or atypical lung resection) that included lymphadenectomies as well, with patients’ medical history available for the clinical data. The samples from biopsies or the ones with insufficient clinical data were excluded from the study. All samples were initially obtained from patients between January 2017 and December 2018. A total of 48 paraffin blocks containing LC samples were collected, 34 of those being ACs, and 14 SCCs. An additional 35 paraffin blocks containing lymph nodes were obtained. From each paraffin block, a 4 μm-thick section was cut, mounted, and dried at 37 °C. Then, the sections were stained with hematoxylin and eosin and re-evaluated by a pathologist.

### 4.2. Immunohistochemical Staining and HSCORE Calculation

Immunostaining was performed on the same serial section of each LC and metastatic lymph node sample as follows: paraffin sections were mounted onto super frost slides (Thermoscientific, Dreieich, Germany) and processed in an automatic stainer (Ventana Bench Mark Ultra autostainer, Ventana Roche, Tucson, AZ, USA). For the detection of GLUT1, primary ‘‘ready-to-use’’ monoclonal mouse antibody (SPM498, Novus Biologicals, Abingdon, UK) was applied in a concentration of 1:100. A UltraView Universal DAB Detection Kit (Ventana, Tucson, AZ, USA) was used as the secondary antibody. The histopathologic analysis was performed manually using a microscope (Olympus BX46 microscope, Tokyo, Japan) and cellSens Standard version 1.9 analysis software (Olympus, Tokyo, Japan). Brown staining of the cell membrane and/or cytoplasm was considered positive. Red blood cells present in the blood vessels of the samples were used as a positive control. The expression of GLUT1 in the samples was determined by the HScore method using the equation HScore = Σ (i + 1) × Pi, where i = intensity of staining with a value of 1 (weak), 2 (moderate), or 3 (strong), and Pi was the percentage of stained LC cells of each intensity [62]. For every LC and metastatic lymph node sample, 10 replicates were analyzed and an HScore was calculated for each of them. The HScore of the entire sample was the arithmetic mean of the HScores of the 10 individual replicates. To decrease inter-observer variations, two histologists analyzed the captured micrographs independently, which yielded an interclass correlation analysis coefficient that was >0.8, indicating excellent an agreement [63].

### 4.3. Immunofluorescent Staining and Signal Quantification

Immunofluorescent staining was also performed, as described previously [64], on the same LC samples. Briefly, tissue samples were deparaffinized in xylene and rehydrated in ethanol solutions of decreasing concentrations, ending with distilled water. Then, the samples were immersed in a sodium citrate buffer (pH 6.0) and heated in a steamer for 30 min. After 5 min of washing in phosphate-buffered saline (PBS), a blocking buffer (ab64226, Abcam, Cambridge, UK) was applied for 30 min. The slides were then incubated overnight at room temperature in a humid chamber with the HIF-1α antibody (mouse monoclonal antibody, sc-13515, Santa Cruz Biotechnology, Inc., Dallas, TX, USA) at a dilution of 1:50. The following day, after two rounds of washing in PBS, the Alexa Fluor^®^488 AffiniPure Anti-Mouse antibody (715-545-150, Jackson Immuno Research Laboratories, Inc., Baltimore, PA, USA) was applied at a 1:300 dilution for one hour in the humid chamber. Three more rounds of washing in PBS followed, after which the DNA-binding solution DAPI (4,6-diamidino-2-phenylindole) was applied for 2 min to stain the nuclei. The slides were washed with distilled water, air-dried, and covered with coverslips using a mounting medium (ImmuMount, Thermo Shandon, Pittsburgh, PA, USA). Omitting primary antibodies from the staining procedure was used to control the specificity of the staining. The specimens were analyzed under a fluorescence microscope (Olympus BX61, Tokyo, Japan) and photographed using a mounted digital camera (Nikon Ri-D2, Nikon, Tokyo, Japan) with NIS-Elements F software version 3.0 (Nikon, Tokyo, Japan). In order to quantify the HIF-1α staining, we analyzed 5 replicates per sample. For each replicate, we counted 100 LC cells and determined the number of those cells that only displayed nuclear HIF-1α staining and either nuclear or cytoplasmatic staining. The mean values of the number of positive cells for each sample were used in further statistical analyses.

### 4.4. Transcriptomics

We retrieved the data for the RNA expression of the *GLUT1* and *HIF-1α* genes from the UCSC Xena database (University of California Santa Cruz, http://xena.ucsc.edu/ (accessed on 19 May 2023)). Overall survival, *GLUT1*, and *HIF-1α* gene expression (mRNA seq) data from the GDC TCGA Lung Adenocarcinoma (LUAD) and GDC TCGA Lung Squamous Cell Carcinoma (LUSC) studies were exported and edited in Microsoft^®^ Excel^®^ 2019 MSO version 2305 (Microsoft Corp., Redmond, WA, USA). After the data curation for double samples, 502 patients were included in the survival analysis for lung adenocarcinomas and 494 patients for squamous cell carcinomas. Survival analysis based on expression groups (i.e., between the lowest and highest 50% for each gene) was completed in GraphPad 9.0.0. software (GraphPad Software, San Diego, CA, USA). The log-rank test and Kaplan–Meier method were used for the statistical analysis of the survival length.

### 4.5. Statistical Data Analysis

All statistical analyses were performed in GraphPad Prism 9.0.0 software (GraphPad Software, San Diego, CA, USA). The normality of the data distribution was checked by the Shapiro–Wilk test. All correlations were performed with Spearman’s rank correlation coefficient. The significance of the differences in GLUT1 expression measured by the HScore methods, HIF-1α protein expression, and FDG uptake measured by SUVmax between sexes, histological subtypes, and PET CT lymph node positivity groups was determined by the Mann–Whitney U test, while the Kruskal–Wallis test with uncorrected Dunn’s post-hoc test were used for the differences between tumor stages. Statistical significance was set at *p* < 0.05. All data are presented as the mean ± standard deviation.

## 5. Conclusions

Based on the results of our study, we conclude that GLUT1 and HIF-1α immunohistochemical expressions influence FDG accumulation, with tumor size being the other and most important factor, and SCCs tend to have higher GLUT1 and HIF-1α expressions than ACs. Additionally, *GLUT1* and *HIF-1α* mRNA expressions could have a prognostic value, depending on the histological subtype; however, further studies are needed to confirm this. On the other hand, PET CT, although a viable diagnostic tool, should be used as a secondary evaluation method when needed and CT should remain the primary imaging test necessary in disease staging.

## Figures and Tables

**Table 1:** Immunohistochemical expression of GLUT1 in primary tumor and lymph node metastasis according to clinical characteristics of patients, lung cancer histology type, and tumor stage.

	GLUT1 Tumor	*p*	GLUT1 Lymph Node	*p*
Male (*n* = 32)	2.71 ± 0.48	0.2896 *	2.74 ± 0.54	0.0577 *
Female (*n* = 16)	2.53 ± 0.39		2.22 ± 0.22	
Adenocarcinoma (*n* = 34)	2.49 ± 0.38	<0.0001 *	2.39 ± 0.37	0.0196 *
Squamous cell carcinoma (*n* = 14)	3.05 ± 0.38		3.22 ± 0.51	
Tumor stage		0.5928 ^†^		0.6545 ^†^
I (*n* = 15)	2.57 ± 0.45			
II (*n* = 11)	2.70 ± 0.37		2.57 ± 0.48	
III (*n* = 18)	2.75 ± 0.50		2.76 ± 0.52	
Lymph nodes		0.1096 *		0.6621 *
PET CT+ (*n* = 11)	2.87 ± 0.40		2.57 ± 0.18	
PET CT− (*n* = 35)	2.59 ± 0.46		2.53 ± 0.58	

* Mann–Whitney U test. ^†^ Kruskal–Wallis test with uncorrected Dunn’s post-hoc test.

**Table 2:** Immunofluorescent expression of HIF-1α in primary tumors according to clinical characteristics of patients, lung cancer histology type, and tumor stage.

	Nuclear HIF-1α	*p*	Nuclear and Cytoplasmatic HIF-1α	*p*
Male (*n* = 12)	51.25 ± 38.33	0.086 *	65.33 ± 26.31	0.0089 *
Female (*n* = 8)	11.00 ± 10.56		26.20 ± 22.85	
Adenocarcinoma (*n* = 10)	14.11 ± 18.16	0.0025 *	34.33 ± 22.02	0.0038 *
Squamous cell carcinoma (*n* = 10)	66.44 ± 30.81		74.33 ± 22.66	
Tumor stage		0.7239 ^†^		0.6266 ^†^
I (*n* = 7)	23.40 ± 36.98		38.20 ± 31.59	
II (*n* = 6)	53.80 ± 44.65		61.00 ± 39.17	
III (*n* = 7)	37.83 ± 35.32		57.83 ± 24.80	
Lymph nodes		0.0121 *		0.0071 *
PET CT+ (*n* = 5)	85.50 ± 2.08		88.50 ± 2.38	
PET CT− (*n* = 15)	25.50 ± 31.89		42.92 ± 28.27	

* Mann–Whitney U test. ^†^ Kruskal–Wallis test with uncorrected Dunn’s post-hoc test.

**Table 3:** SUVmax values in primary tumor and lymph nodes according to clinical characteristics of patients, lung cancer histology type, tumor stage, and lymph node involvement based on PET CT.

	Tumor SUVmax	*p*	Lymph Node SUVmax	*p*
Male (*n* = 32)	7.81 ± 4.36	0.4778 *	5.41 ± 1.78	0.3455 *
Female (*n* = 16)	8.86 ± 4.82		4.37 ± 1.51	
Adenocarcinoma (*n* = 34)	8.20 ± 4.27	0.6909 *	6.05 ± 1.84	0.2182 *
Squamous cell carcinoma (*n* = 14)	8.05 ± 5.18		4.60 ± 1.51	
Tumor stage		0.2683 ^†^		0.515 ^†^
I (*n* = 15)	7.16 ± 4.18		3.50 ± 0.28	
II (*n* = 11)	8.40 ± 6.12		4.60 ± 1.55	
III (*n* = 18)	9.17 ± 3.83		5.93 ± 1.96	
Lymph nodes		0.055 *		
PET CT+	9.80 ± 5.51			
PET CT−	7.33 ± 3.74			

* Mann–Whitney U test. ^†^ Kruskal–Wallis test with uncorrected Dunn’s post-hoc test.

## Data Availability

Data regarding the expression of GLUT1 and HIF-1α in lung carcinomas can be found at the publicly available website http://xena.ucsc.edu/ which contains databases for gene expression in cancers.
